# Editorial: Futsal Research and Challenges for Sport Development

**DOI:** 10.3389/fpsyg.2022.856563

**Published:** 2022-03-21

**Authors:** Cesar Méndez-Dominguez, Fábio Y. Nakamura, Bruno Travassos

**Affiliations:** ^1^Department of Physical Education and Sports, Rey Juan Carlos University, Madrid, Spain; ^2^Department of Didactics of Languages, Arts and Physical Education, Complutense University of Madrid, Madrid, Spain; ^3^Research Center in Sports Sciences, Health Sciences and Human Development (CIDESD), University of Maia (ISMAI), Maia, Portugal; ^4^Department of Sport Sciences, Research Center in Sports Sciences, Health Sciences and Human Development (CIDESD), Universidade da Beira Interior, Covilhã, Portugal; ^5^Portugal Football School, Portuguese Football Association, Federação Portuguesa de Futebol (FPF), Covilha, Portugal

**Keywords:** futsal, performance, learning process, monitoring and testing, player development, coaching (performance)

Futsal is an indoor team sport that is played worldwide by men and women, boys and girls, in both professional and amateur leagues but also in schools and most of futsal academies as a strategy to improve players' development (Barbero-Alvarez et al., 2008). Over the last 15 years its popularity increased a lot, which may be confirmed by the rising number of male and female participants, and it is being promoted and recognized by FIFA and UEFA, with the organization of new youth and senior female European Competitions (Lago-Fuentes et al., 2020). Although futsal is one of the sports with the greatest increase in the number of practitioners and social recognition worldwide, both recreationally and competitively, in recent years, such development has not been accompanied by a similar number of investigations that support the intervention of the coach. Thus, in this Research Topic we invited the submission of manuscripts that promoted the transfer of theoretical research and contribute with practical implications for the development of futsal.

From the overall of the twenty-six manuscripts submitted and twenty-two accepted, nine (40%) were led by Spanish authors, and seven (32%) by Brazilian authors. The other submissions came from Portugal and Taiwan (9% each), and from Croatia and Japan (4.5% each). Of the total of twenty-two accepted manuscripts, fourteen were focused on the physical and physiological futsal demands, in addition to 1 systematic review that includes common aspects; another 2 aimed to technical-tactical aspects, 2 regarding different aspects of performance in female futsal players, and another 3 related to psychological and biomechanical aspects.

The most relevant topic in our compilation was related with the characterization of the physical and physiological demands of futsal performance, through the measurement of internal and external load indicators in training and competition. Seven manuscript were focused in external load: (i) Illa, Fernandez, Reche, Carmona, et al., using a Local Positioning System (LPS) [similar technology to a Global Positioning System (GPS), but which only provides players position information relative to a local field or area], carried out an accurate evaluation of the external load demands in competition and training, describing the frequency and distribution throughout the weekly microcycle to find high and very high demand scenarios that coincide with matches, but also with the training session held 2 days before the match; (ii) also using the same system, Fernandez, Reche, Carmona, Serpiello, et al. quantified the most demanding external load scenarios in elite futsal matches, identifying differences between playing positions (defenders, wingers, and pivots) during different time windows, and also between matches, suggesting the importance of contextual variables like conditioning factors of the “requirement degree”; (iii) Silva et al. focused on the external load variables during pre-match warm-up routines, since the tasks in which intensity increases mainly due to the greater number of accelerations and decelerations per minute; (iv) with the main goal of match demands characterization, Ribeiro et al. highlighted the importance of deceleration in order to cope with match situations and master the space-time relationship to gain advantage, which makes the accelerations and decelerations variables the ones discriminating elite futsal players; (v) Sekulic et al. analyzed the importance of the futsal specific capacities and abilities to differentiate performance levels between professional players, and those who are starters and substitutes; and (vi) Garcia-Unanue et al. identified performance differences in favor of elite players compared to non-elite players in sprint and agility tests, although the contractile properties of the lower body was not a determining factor.

The last manuscript of this group analyzed the physical demands of elite futsal referees during competition. Serrano et al. monitored with the Local Positioning System (LPS) the physical demands of elite referees to provide knowledge of the specific activity profile during futsal matches. In line with previous research with futsal players', the characterization of physical demands of futsal referees can be useful to design suitable training programs.

Focusing on the variables of internal load, seven manuscripts were accepted: (i) Dos-Santos et al. measured % HR (Heart Rate) mean and blood Lactate Concentration [La-] and verified that balancing the number of substitutions and the players permanence on the court in both halves promoted similar intensities between the 1st and the 2nd half; (ii) Campos et al. compared the use of two models of High Intensity Interval Training (HIIT) in order to achieve a better training prescription in young futsal players, according to game demands; (iii) Chen et al. measured very short-term heart rate variability records to provide information on training adaptation and autonomic nervous system recovery status in under 20 elite futsal players; (iv) Silva et al. considered the weak correlation between VO_2_ estimated from the relationship with HR on a treadmill and VO_2_ measured in a simulated futsal game and its poor agreement linked to the futsal intermittent nature; (v) Lu et al. used the perceived measures of training load at the respiratory (respiratory Rating Perceived Exertion RPE) and muscular (muscular Rating Perceived Exertion RPE) levels and cardiac responses in association with training load as useful indicators to understand the young futsal players demands; and (vi) Stochi de Oliveira verified the significant differences established between the competitive and the preparatory periods of the season in the Internal Training Load (ITL) computed through the relationship between the training volume and the Rating Perceived Exertion (RPE). (vii) In line with previous analysis of RPE, Polito et al. analyzed the construct validity of a specific pictorial scale of perceived exertion (GOAL Scale), in which the absence of verbal anchors makes it possible to use it from an inclusive point of view for football, futsal and beach soccer players in different languages and different levels of literacy.

In addition, as a finishing touch to highlight the importance of the biological condition of the players for performance in futsal, the systematic review carried out by Spyrou et al. highlighted the demands of futsal considering different game dimensions such as: internal and external load data, physiological, neuromuscular, and biochemical responses. They also screen the studies to find differences in variables linked to elite and non-elite futsal competitions.

In fact, the description of futsal game demands by considering new technological systems (e.g., tracking systems such as Local Positioning Systems, LPS; video tracking) to measure external and internal load variables in highly competitive environments and during training sessions should be promoted in the future in order to fill the gap between research and practice and promoting more adjusted performance indicators and metrics according to the specific demands of futsal (Ribeiro et al.).

The collection could not miss the offensive and defensive tactical aspects of futsal teams and players by controlling variables associated with the game that lead to the scoring goal or trying to avoid it. Two manuscripts were accepted, one focused on the offensive aspect, while another on the defensive action. Amatria et al. analyzed the construction of offensive sequences, using sequential analysis of delays as diachronic analysis, to identify the game patterns that end with a goal scored in two of the main European futsal leagues (Spanish and Italian); Pizarro et al. analyzed the indirect effects of an intervention program based on a Non-Linear Pedagogy approach (NLP) through the use of Small-Sided and Conditioned Games (SSCG) on Decision-Making (DM) and Executions (Ex) relative to defensive tactical behaviors carried out by young futsal players.

Although the low number of studies submitted in this field, further research should be developed focused on the technical-tactical aspects of the match due it is a decisive aspect of performance (Méndez et al., 2019), and the one that tends to occupy more workload in futsal training. In addition, further studies that combine physiological and technical-tactical variables are required in order to further contextualize the specific game demands of futsal, as it occurs over the match and following the coaches' perspectives. As previously mentioned, the use of new technological systems (e.g., tracking systems such as Local Positioning Systems, LPS; video tracking) to capture positional data of players and teams should be considered to access to spatial-temporal relations between players and teams that sustain specificities of the futsal match in different game environments (Travassos et al., 2013) and in different levels of practice (Travassos et al., 2018).

The inclusion of elite female futsal players in the research studies was an excellent new as a distinctive feature within this Research Topic. First, Cejudo et al. determined the lower extremities joints Range Of Motion (ROM) profile in futsal players by sex, position, competitive level and bilaterality, listing the possible differences between female players with their male counterparts and the implications at the level of physical performance. Second, Queiroga et al. aimed to characterize the age of onset of training, age at menarche, menstrual periodicity, and performance perception during the menstrual cycle, and examined the impact of these reproductive variables on body composition, morphology (somatotype), and body weight satisfaction in Brazilian elite futsal players.

Research developed with female futsal players should be encouraged in order to better characterize it's specificities, uncovering the match demands and improving coaches' practice.

Regarding injuries and mechanical aspects of performance, Rúiz-Pérez et al. applied a supervised Machine Learning techniques to predict Lower Extremity non-contact Soft Tissue (LE-ST) injuries, allowing better identification of elite futsal players at higher risk. Finally Ismail et al. investigated the possible influence of forefoot bending stiffness property of three commercial futsal shoes on change of direction run resultant performance.

Further development of epidemiological studies developed longitudinally and in different levels of practice and sex are required to improve the strategies for the evolution and improvement of players' capacities and injury prevention.

Finally, only one study considered the analysis of psychological aspects in futsal. Brandão et al. used the exploratory Bi-factorial model (BI-ESEM) to evaluate the list of stressors in elite futsal through a self-report instrument, where they tried to know the impact of stressful situations at the level of practical implications for psychological intervention and for improvement of player's life.

Regarding psychological aspects that characterizes and determines performance of futsal players, limited knowledge exists related with this sub-discipline that sustain players' and teams' performance. For sure, it will be a hot topic for the future with great impact in practice.

In light of the great number of high-quality manuscripts published, this special issue has made an important contribution to the development of knowledge in futsal. For the next years, we expect that the current research could be the basis to improve the understanding about physical, technical and tactical game demands, but also to sustain the development of new research that addresses practical implications, particularly related with coaching, pedagogy, talent development, performance analysis, motion analysis, and psychological intervention.

## Author Contributions

All authors have contributed to the preface, summary and epilog of this editorial article and to its subsequent revision. All authors contributed to the article and approved the submitted version.

## Conflict of Interest

The authors declare that the research was conducted in the absence of any commercial or financial relationships that could be construed as a potential conflict of interest.

## Publisher's Note

All claims expressed in this article are solely those of the authors and do not necessarily represent those of their affiliated organizations, or those of the publisher, the editors and the reviewers. Any product that may be evaluated in this article, or claim that may be made by its manufacturer, is not guaranteed or endorsed by the publisher.

## References

[B1] Barbero-Alvarez J. C. Soto V. M. Barbero-Alvarez V. Granda-Vera J. (2008). Match analysis and heart rate of futsal players during competition. J. Sports Sci. 26, 63–73. 10.1080/02640410701287289 17899472

[B2] Lago-Fuentes C. Jimenez-Loaisa A. Padron-Cabo A. Fernandez-Villarino M. Mecias-Calvo M. Travassos B.. (2020). Monitoring workloads of a professional female futsal team over a season: a case study. Sports 8, 69. 10.3390/sports8050069 32438612 PMC7281136

[B3] Méndez C. Santos J. Ribeiro J. N. Gonçalves B. Travassos B. (2019). Attacking profiles of the best ranked teams from elite futsal leagues. Front. Psychol. 10, 1370. 10.3389/fpsyg.2019.01370 31281280 PMC6596354

[B4] Travassos B. Coutinho D. Gonçalves B. Pedroso P. Sampaio J. (2018). Effects of manipulating the number of targets in U9, U11, U15 and U17 futsal players' tactical behaviour. Hum. Mov. Sci. 61, 19–26. 10.1016/j.humov.2018.06.017 30005844

[B5] Travassos B. Davids K. Araujo D. Esteves P. (2013). Performance analysis in team sports: advances from an ecological dynamics approach. Int. J. Performance Anal. Sport 13, 83–95. 10.1080/24748668.2013.11868633

